# The unique distribution of the *Plasmodium vivax* merozoite surface protein 1 in parasite isolates with short and long latent periods from the Republic of Korea

**DOI:** 10.1186/s12936-015-0803-3

**Published:** 2015-08-05

**Authors:** Youn-Kyoung Goo, Jun-Hye Moon, So-Young Ji, Dong-Il Chung, Yeonchul Hong, Shin-Hyung Cho, Won-Ja Lee, Jung-Yeon Kim

**Affiliations:** Division of Malaria and Parasitic Diseases, National Institute of Health, Korea CDC, Osong Saeng-myeong 2 ro, Osong Health Technology Administration, Osong, Republic of Korea; Department of Parasitology and Tropical Medicine, Kyungpook National University School of Medicine, Daegu, 700-422 Republic of Korea

## Abstract

**Background:**

Vivax malaria occurring in the Republic of Korea is occasionally characterized by a long latent infection induced by hypnozoites in the liver. So far, the mechanisms responsible for short and long latent infections of vivax malaria are not known. Therefore, the present study classified the parasite isolates according to the long and short latent periods and then analysed the genetic diversity of the *Plasmodium vivax* merozoite surface protein 1 (PvMSP-1).

**Methods:**

Blood samples containing *P. vivax* isolates were collected from 465 patients from 2011 to 2013 at health centers in the Republic of Korea. PvMSP-1 gene sequences were analysed in groups classified by the collection year, and short or long latent periods. The samples in short and long latent periods were selected by the timing of vivax malaria occurrence, July–August and January–May, respectively.

**Results:**

Three PvMSP-1 types (Sal-1, Belem, and recombinant) were observed in *P. vivax* isolates collected from 2011 to 2013. Interestingly, the recombinant and Sal-1 types were dominant in vivax malaria of the long and short latent periods, respectively. In addition, the S-b like subtype of the PvMSP-1 Sal-1 type was first identified in 2013.

**Conclusion:**

This study revealed that the genetic type of PvMSP-1 is likely related to the duration of its latent period. Moreover, trends of the genetic types of PvMSP-1 seem to be stable in recent years compared with those of previous years in which various new types were observed.

## Background

Vivax malaria caused by *Plasmodium vivax* is the most prevalent form of human malaria and afflicts several hundred million people annually [1]. It is endemic in tropical and subtropical countries of Africa, the Middle East, the South Pacific, Central and South America, and Asia, including the Republic of Korea (ROK) [2]. In the ROK, *P. vivax* infection was highly endemic and considered an indigenous disease by the late 1970s. Subsequently, vivax malaria was eradicated by efforts of the World Health Organization and the National Malaria Eradication Programme, until the disease was found in a soldier serving in the demilitarized zone (DMZ) between the ROK and the Democratic People’s Republic of Korea (DPRK) in 1993 [3, 4]. Since then, the number of vivax malaria cases reached more than 4,000 cases in 2000, decreased again to less than 500 cases in 2013, and increased to approximately 670 cases in 2014 [5]. The occurrence of vivax malaria is concentrated in regions close to the DMZ; thus, it has been suggested that the outbreaks of vivax malaria in the ROK and the DPRK are correlated [6, 7].

Vivax malaria transmission in the ROK is unstable and seasonal, peaking in July and August because the climate is temperate [8, 9]. Compared to *Plasmodium falciparum*, *P. vivax* infection is characterized by a relapse caused by a hypnozoite in the human liver [10, 11]. After transmission via a bite from a *P. vivax*-infected mosquito, sporozoites generally develop into schizonts that result in the primary illness. However, some sporozoites enter hepatocytes and then develop into hypnozoites in the human liver [12, 13]. The hypnozoites can become active months or even years later, leading to a relapse of vivax malaria after resolution of the primary illness [14]. The mechanisms responsible for the development of the hypnozoite stage and the subsequent relapse of the disease are still unknown. In addition, the presence of hypnozoites results in a prolonged incubation period of vivax malaria referred to as a long latent period, while vivax malaria parasites generally lead to primary illness after a short latent period (within several days after a vivax malaria-infected mosquito bite) [15]. Although the occurrence of vivax malaria in the ROK is seasonal in July–August (summer season), a few cases are reported during winter season as a result of parasites with long latent period.

Genotyping studies have been used to gain a better understanding of the population structure of pathogens, including the *Plasmodium* species, and to develop an effective vaccine against these parasites [16–18]. The population structure of certain genes could explain the transmission dynamics and evolutionary trend of the *Plasmodium* species. Moreover, genetic diversity of vaccine candidates has been investigated because the extensive genetic diversity within natural malaria parasite populations has hampered efforts to develop an effective vaccine [19, 20]. Merozoite surface protein 1 of *P. vivax* (PvMSP-1) is a well-known vaccine candidate as well as a marker for genotyping [21–23]. PvMSP-1 is composed of several variable blocks flanked by 10 conserved regions containing a dimorphic pattern. Block 5 is located between two of the interspecies conserved blocks (ICBs), ICB5 and ICB6, and shows a dimorphic pattern of sequences with little homology. The first two isolates of polymorphic PvMSP-1 were identified in Latin America and named the Brazilian Belem (Belem) and Salvador (Sal-1) strains [24, 25]. Shortly after, the mosaic organization and heterogeneity in the frequency of allelic recombination of PvMSP-1 were demonstrated in *P. vivax* isolates from different countries [26]. The results of these analyses of PvMSP-1 polymorphisms have enhanced the data collected from epidemiological and molecular biological surveys.

In the present study, the sequences of PvMSP-1 in *P. vivax* isolates collected from 2011 to 2013 in the ROK were analysed. The sequence data were evaluated to understand the genetic diversity of the recent *P. vivax* population in the ROK in comparison with that of previously studied populations [27, 28]. In addition, the genotypes of vivax malaria parasites involved in the long and short latent periods were analysed.

## Methods

### Ethics

The study was approved by the ethics committee of the Korea National Institute of Health. An approval form was used to obtain written informed consent from each participant. Each participant also consented to provide a 5-mL blood sample.

### Sample collection

Venous blood samples preserved with EDTA were received from 465 patients diagnosed with vivax malaria at local health centers and hospitals from 2011 to 2013. Eighty-five, 248, and 132 samples were collected in 2011, 2012, and 2013, respectively. Microscopic examination of Giemsa-stained thick and thin blood films was used to confirm the diagnosis. Blood specimens were kept cool and arrived at the Korea Centers for Disease Control and Prevention (KCDC) within 24 h after collection. Long or short latent period groups were classified using an arbitrary definition according to the date of the patient’s visit to the hospital with malaria symptoms. The short and long latent periods of vivax malaria were defined as a hospital visit date in July–August and January–May, respectively.

### DNA extraction

Genomic DNA of *P. vivax* was extracted from 200 μL of each blood sample using a QIAamp DNA Mini Kit (Qiagen, USA) following the manufacturer’s instructions.

### PCR amplification and sequencing of PvMSP-1

The sequence between ICBs 5 and 6 of MSP-1 of *P. vivax* was amplified as previously described [27]. Primers used for the amplification were MSP1A (5′-GAGCCCTACTACTTGATGGTCC-3′) and MSP1B (5′-CCTTCTGGTACAGCTCAATG-3′). Amplification was performed in a 20-μL reaction mixture containing the genomic DNA of *P. vivax*, 1 × reaction buffer, 2.5 mM MgCl_2_, 0.2 mM of each dNTP, 10 pmol of each primer, and 1 unit of Ex Taq DNA polymerase (Takara, Japan). PCR cycling conditions included an initial denaturation at 95°C for 7 min; followed by 35 cycles of denaturation at 94°C for 1 min, annealing at 58°C for 1 min, and extension at 72°C for 1 min; and a final extension at 72°C for 7 min. The PCR products were subjected to electrophoresis on a 1% agarose gel.

### Purification of PCR products and sequencing of PvMSP-1

The PCR products were purified using a QIAquick Gel Extraction kit (Qiagen, USA) according to the manufacturer’s instructions. After gel purification, the PCR products were sequenced with the primers from ICB5–ICB6 using an ABI 3730XL DNA Analyzer automated sequencer (Applied Biosystems, USA).

### Sequence analysis

Parasite gene sequences obtained from the blood samples of the malaria patients in this study were compared with previously published PvMSP-1 gene sequences. CLC Sequence Viewer 6.8.1 (CLC bio, USA) was used to convert nucleotide sequences into amino acid sequences and to align the sequences.

### Statistical analysis

Any statistically significant differences were determined by Student’s *t* test using JMP Version 8 (SAS Institute Inc., USA). *P* < 0.05 was considered to be statistically significant.

## Results

**Fig. 1:**
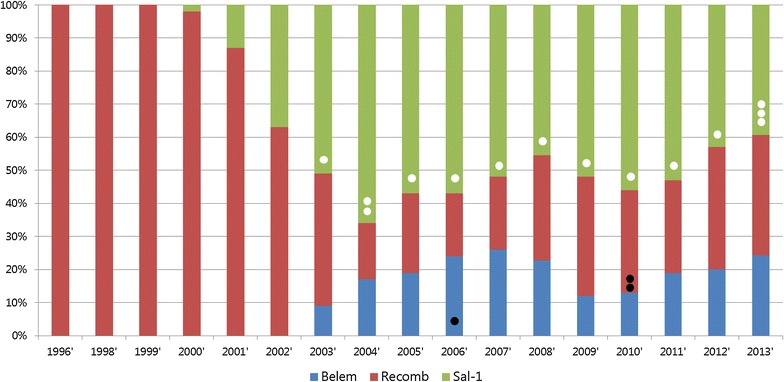
Frequency of *Plasmodium vivax* merozoite surface protein 1 (PvMSP-1) strains in the ROK during the previous (1996–2010) and current (2011–2013) study periods. No spot regions indicate only one subtype (S-b, B-1, and R-1) without variation; *single open circle* S-a subtype, *double open circle* S-a and S-c subtypes, *triple open circle* S-b like subtype, *single* *filled circle* B-2 subtype, *double filled circle* R2, R3, and R4 subtypes.

The polymorphisms within block 5 of PvMSP1 were analysed from 465 isolates collected from 2011 to 2013. Three PvMSP-1 types (Sal-1, Belem, and recombinant types) have been found previously in Korean vivax isolates. Since 2003, the majority of isolates have belonged to the Sal-1 type; however, the proportion of Sal-1 isolates has been decreasing each year since 2011 (Fig. 1). As shown in Table 1, the percentage of Sal-1 isolates in each year was 52.9% (2011), 42.7% (2012), and 39.4% (2013). Since 2004, both the S-a and S-b subtype isolates have coexisted in the ROK. Recently, isolates of the S-b subtype of Sal-1 were found to have decreased from 24.7% in 2011 to 15.2% in 2013, while the S-a subtype showed a similar decrease in proportion among *P. vivax* isolates (28.2% in 2011, 24.2% in both 2012 and 2013). Interestingly, one isolate of the S-b type harbored a mutation that changed a glycine residue to a glutamic acid residue at position 725 (p.G725E). Thus, this isolate was named as the S-b like subtype. In comparison with Sal-1 type isolates, the number of PvMSP-1 Belem type isolates has been increasing from 2011 to 2013. All isolates of the Belem type were of the B-1 subtype. The percentage of PvMSP-1 recombinant type isolates in 2012 (37.1%) and 2013 (36.4%) was increased compared to that seen in 2011 (28.2%). Although the R2, R3, and R4 subtypes of the PvMSP-1 recombinant type were found in 2010, these subtypes did not appear from 2011 to 2013. During the three-year period, only the R1 subtype was observed.

**Table 1:** PvMSP-1 block 5 allelic subtype frequencies of *P. vivax* isolates from the Republic of Korea

Year	No. of samples (%)				
	Recombinant	Belem	Sal-1		Total
		B-1	S-a	S-b	
2011	24 (28.2)	16 (18.9)	24 (28.2)	21 (24.7)	85
2012	92 (37.1)	50 (20.2)	60 (24.2)	46 (18.5)	248
2013	48 (36.4)	32 (24.2)	32 (24.2)	20 (15.2)	132

**Fig. 2:**
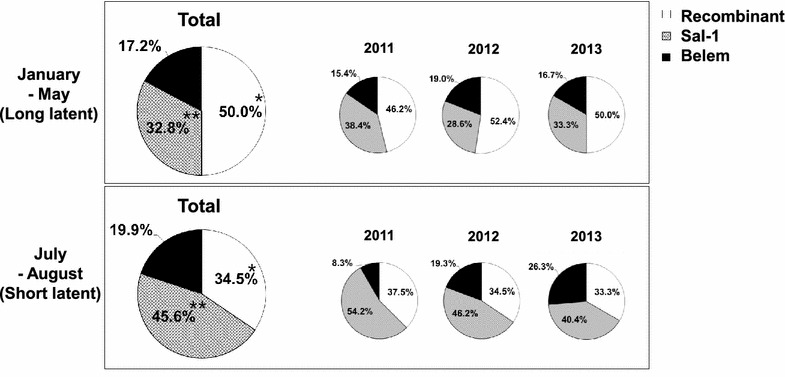
Frequency of *Plasmodium vivax* merozoite surface protein 1 (PvMSP-1) strains in the ROK in the long (January–May) and short (July–August) latent periods from 2011 to 2013. *significant difference (*P* < 0.05) in the PvMSP-1 recombinant type proportion between long and short latent periods; **significant difference (*P* < 0.05) in the PvMSP-1 Sal-1 type proportion between long and short latent periods.

Each sample was subsequently classified into long or short latent period groups according to the date of the patient’s visit to the hospital with malaria symptoms. The short and long latent periods of vivax malaria were defined by a hospital visit date in July–August and January–May, respectively. Of the original 465 samples, 226 were selected for the short latent period of vivax malaria: 24 from 2011, 145 from 2012, and 57 from 2013. There were 58 total samples from the long latent period from 2011 (n = 13), 2012 (n = 21), and 2013 (n = 24). The PvMSP-1 types in both periods showed the same pattern in each year: the MSP-1 recombinant type was significantly predominant in the long latent period samples (*P* < 0.05), while the MSP-1 Sal-1 type was most prevalent in the short latent period samples (*P* < 0.05). As shown in Fig. 2, the PvMSP-1 recombinant type accounted for 46.2% (2011), 52.4% (2012), and 50.0% (2013) of the long latent period isolates (January-May), but only 37.5, 34.5, and 33.3% of the short latent period samples (July–August) from each respective year. However, the proportions of the PvMSP-1 Sal-1 type, 38.4% in 2011, 28.6% in 2012, and 33.3% in 2013, in the long latent period isolates were increased to 54.2, 46.2, and 40.4% in the short latent period isolates of each respective year (Fig. 2).

## Discussion

In the current study, the genetic diversity of the *P. vivax* merozoite surface protein 1 gene was investigated in field isolates from vivax malaria patients in a high transmission area of the Republic of Korea (ROK) as a continuation of two previous reports [27, 28]. In order to compare the genetic structure of the ROK isolates, sequences from Block 5 were analysed, a variable region of PvMSP-1. After the PvMSP-1 Sal-1 type and Belem type were first reported in 2000 and 2003, respectively, all three strains of *P. vivax* (Sal-1, Belem, and recombinant types) were found in 2011–2013. Interestingly, a PvMSP-1 S-b like type, containing p.G725E, which changes glycine to glutamic acid, was found in an isolate collected in 2013. It seems likely that genetic recombination resulting in a single nucleotide polymorphism (SNP) occurred in the PvMSP-1 gene, because PvMSP-1 is a highly polymorphic antigen [29]. Moreover, the *P. vivax* recombinant type increased in the later years (2012 and 2013) compared with 2011, which is likely a result of genetic recombination or a crossing of *P. vivax* Sal-1 and Belem types. However, co-infection of PvMSP-1 Sal-1 and Belem types in a single sample was not observed, which could have indicated the possibility of crossing of the *P. vivax* types. That might be due to the fact this genotyping that used simple PCR methods and followed sequencing analysis tends to amplify only the dominant type. Therefore, further polyclonal infection studies should be done using microsatellite or pyrosequencing analysis, which are known to be capable of identifying mixed infections. In addition, only the original PvMSP-1 recombinant type (10×Q) appeared during the three-year period (2011–2013), whereas high levels of polymorphism in the number of Q repeats were observed in 2010 (12×Q, 14×Q, and 27×Q). Although the regions of sample collection were the same, the new subtypes with different numbers of Q residue repeats were not observed during the recent sample collection period. This indicates that the 12×Q, 14×Q, and 27×Q subtypes did not become established and subsequently disappeared from the *P. vivax* population of the ROK, like the S-c and B-2 subtypes only in 2003 and 2006, respectively. This also suggests that transmission might not be dynamic in the ROK. This finding was supported by a microsatellite study of ROK isolates suggesting continuous introduction of *P. vivax* variants into the ROK from other parasite population sources, especially from DPRK [30, 31].

Vivax malaria in the ROK is characterized by a long latent infection. The incubation period of a primary infection of vivax malaria is 7–30 days; however, in the ROK, the disease sometimes does not show an onset of symptoms until several months after the initial infection by mosquito bites [32]. Therefore, in the present study, samples were divided into short (July–August) and long (January–May) latent periods using an arbitrary definition. And then, *P. vivax* isolates were analysed in order to determine the dominant strains involved in both the short and long latent periods of vivax malaria. To date, the major *P. vivax* strains inducing the short or long latent periods of vivax malaria have not been investigated. This study, using field isolates of the short and long latent periods, showed that *P. vivax* of the PvMSP-1 recombinant type was dominant in the long latent period isolates. This finding was consistent over three successive years (2011–2013). Based on this result, it is likely that this specific *P. vivax* strain easily induces long latency and the development of the hypnozoite. However, further broad surveillance studies would be necessary in order to verify this finding.

The merozoite surface protein 1 is one of the most studied vaccine candidates of vivax malaria. High genetic polymorphism within PvMSP-1 has been reported in other countries. In addition, the genetic diversity has increased over the past decade from two genotypes to six found in the ROK. This allelic polymorphism is one of the greatest hurdles to inducing a protective immune response against the genetically diverse *P. vivax* because amino acid variations can affect the immunogenic properties of antigens. Therefore, continuous research will be necessary to determine the impact of antigenic diversification on the immunogenicity of the PvMSP-1 antigen in natural populations.

## Conclusion

Sequence analysis of Block 5 of PvMSP-1 identified the Sal-1 (S-a and S-b subtypes), Belem (B-1 subtype), and recombinant types in *Plasmodium vivax* isolates collected from the Republic of Korea from 2011 to 2013. The trends of the genetic types of PvMSP-1 seem to be consistent with previous years, except for the appearance in 2013 of an S-b like subtype of the Sal-1 type containing one SNP. Interestingly, the PvMSP-1 recombinant type and the Sal-1 type were the predominant types observed in *P. vivax* isolates in the long and short latent periods, respectively.
